# Czech Cheeses: Their History, Production, and Chemical and Sensory Qualities

**DOI:** 10.1111/1750-3841.70261

**Published:** 2025-05-07

**Authors:** Sandra Teresita Martín‐del‐Campo, Alexa Pérez‐Alva, Diana Karina Baigts‐Allende

**Affiliations:** ^1^ DRIFT‐FOOD Research Centre, Faculty of Agrobiology, Food and Natural Resources Czech University of Life Sciences Prague Prague Czechia

## Abstract

This study presents a comprehensive overview of the most well‐known Czech cheeses. It starts with an overview of the three Protected Geographical Indication (PGI) cheeses, followed by a summary of other traditional or well‐appreciated cheeses. The cheese‐making industry has experienced constant growth, and European cheeses are appreciated globally and locally. The Czech cheese industry is not the exception. However, despite the Czech cheese‐making tradition, there is a dearth of scientific knowledge. Most information is outdated, and access to historical databases is insufficient. For the Czech cheeses evaluated in this study, the cheese‐making process, alongside the available compositional and functional information, is described when possible. The PGI Olomoucké tvarůžky, Jihočeská Niva, Jihočeská Zlatá Niva cheeses, and the non‐protected Blat'ácke zlato cheese have more extensive scientific information presented. Other traditional Czech cheeses, such as Romadur, Nalžov, or Sázava, could be granted a PGI if research provides deeper knowledge about the impact of culture, production region, processes, and ripening, among others, in their unique characteristics.

## 1 Introduction

The history of cheese‐making is thoroughly documented, with origins in the Fertile Crescent, particularly in the Anatolia region (Kindstedt 2012), where the presence of milk fat in pottery shards from the 6500–6000 BC archeological strata indicates the earliest evidence of cheese production (Kindstedt 2012; Salque et al. 2013).

The Neolithic expansion spread cheese‐making first to Turkey and the Balkan Peninsula, and then along the Danube and Rhine rivers. By 4500 BC, the Neolithic Near East culture had a dominant presence in most of Europe, and cheese‐making had reached the North Sea (Kindstedt 2012). In Central Europe, archeological findings in the Linear Pottery settlements along the lower Vistula River, particularly in Kuyavia (Poland), demonstrate the presence of ruminant dairy fat in pottery sieves and cooking pots from 5400 to 4800 BC (Salque et al. 2013). The tradition of cheese production in the Czech lands dates back centuries, with origins in the Kingdom of Bohemia. Initially, Czech cheeses were produced for self‐consumption as farmer cheeses. The development of the railway in the mid‐19th century marked a significant milestone in the evolution of cheese‐making. The most traditional and original Czech cheese is the Olomoucké tvarůžky (Hybšová 2020). The origins of this cheese can be traced back to the Hana lowland in the Moravia Region, situated around Olomouc, during the late 15th century to the early 16th century (Bubík 2015; European Union 2007). The product gained a high reputation at the end of the 19th century, as evidenced by its award at the first Austrian Dairy Exhibition in Vienna in 1872 (A. W. spol. s r. o. 2024; European Union 2007). Nalžovy is a Czech cheese that is similar to Camembert. It has been produced in the south of the Czech Republic since the late 19th century (Mrázek et al. 2016).

The Czech Republic is one of the Central European territories of the former socialist Europe. This communist past was marked by the collectivization of the dairy sector (Ricard 2016). In the mid‐1940s, the socialist economy underwent a significant restructuring of the dairy sector, encompassing both upstream and downstream operations. This restructuring led to the establishment of state farms and cooperatives, marking a departure from the traditional peasant production system. Private dairies were collectivized to establish new state‐owned industrial dairies (Ricard 2016). This restructuring led to the establishment of state farms and cooperatives, marking a departure from the traditional peasant production system. Private dairies were collectivized to establish new state‐owned industrial dairies. The new production model was based on the production volume of ordinary and standard products that were not connected to tradition, such as generic cheeses (Ricard 2016). The production of Niva cheese commenced at this time. Since 1951, the production of Niva cheese has been carried out at the plant in Český Krumlov, employing the same process until the present day (Kružíková and Lakdawala 2024).

After the 1989 revolutions, collectivization began, and in the former Czechoslovakia, the operating structures were mostly maintained. The capital of the collective farms was distributed to the cooperative members, who were subsequently reorganized as companies. In many cases, the new units did not find their place in the market since they were outdated, too small, or poorly managed (Ricard 2016). The arrival of foreign enterprises between 1992 and 1993 had a significant impact on the sector since they established a strong presence between 1995 and 2005, that is, Italian firms based in Litovel, near Olomouc, established a production facility to produce parmesan‐type cheese called Grand Moravia (Ricard 2016). Still, some enterprises with national capital continued working to be next privatized, such as OLMA (Olomouc region). Other enterprises founded before socialism, such as MADETA (České Budějovice), continue producing traditional cheeses.

**FIGURE 1:**
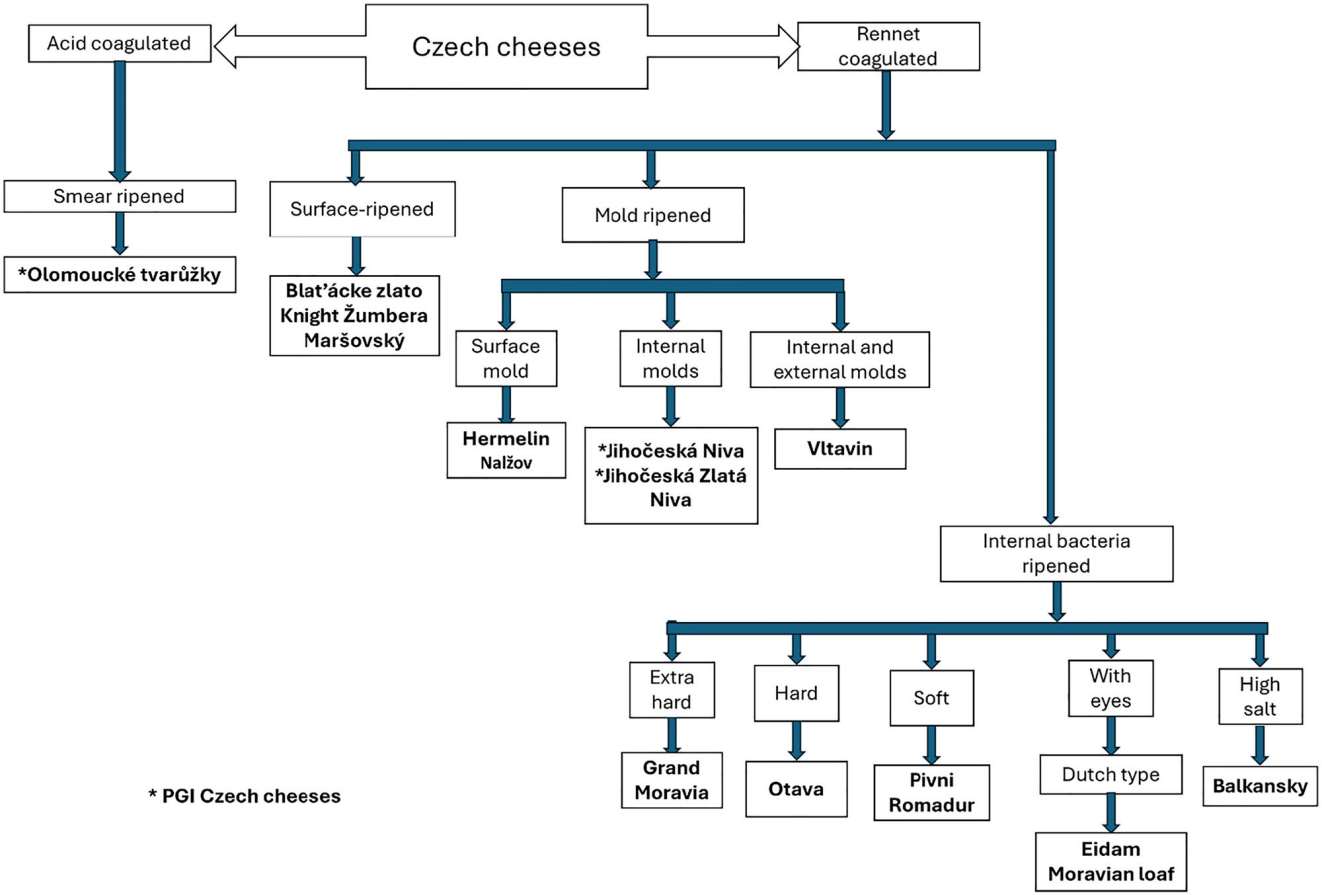
Diversity of Czech cheeses. Classification is according to the coagulation method, and subclassification is according to the principal ripening agents.

Despite these historical aspects, many local and traditional Czech cheeses are still consumed locally and are not of interest to large enterprises or scientific research. The superior quality of most of these local products is likely attributable to a number of factors including unique production methods, their characteristics, and the specific geographic region, making it possible to differentiate their products from similar ones (Albuquerque et al. 2018). Figure 1 presents a classification of the most well‐known Czech cheeses according to the coagulation process. This study aims to recover the scarce information about traditional Czech cheeses, including their composition and properties, in a compiled document, starting with the Protected Geographical Indication (PGI) cheeses and presenting other highly appreciated non‐protected Czech cheeses.

## 2 Protected Geographical Indication Cheeses

Aiming to protect the name of some products, the European Union could grant a “Protected Geographical Indication” (PGI) when they are linked to a place where they are made (European Commission 2024). This protection is linked to the names of these specific products, protecting their unique characteristics linked to their geographical origin and traditional know‐how (European Commission 2024). The PGI helps the producers not only to increase their quality but also to gain consumers' trust in their exceptional quality.

In 2010, three cheeses from the Czech Republic were granted a PGI status (European Commission 2024). Those are the Olomoucké tvarůžky (Olomouc Cheese), Jihočeská Niva (South Bohemian Niva Cheese), and Jihočeská Zlatá Niva (South Bohemian Golden Niva Cheese; European Commission 2024; European Union 2010a, 2010b, 2010c; MZE 2024).

### 2.1 Olomoucké Tvarůžky

#### 2.1.1 PGI and Production Process

Olomoucké tvarůžky (traditionally referred to as tvarůžková) cheese or Olomouc cheese is the only autochthonous Czech cheese that has been granted a PGI (Hybšová 2020; Hanušová et al. 2010). This cheese is produced exclusively in the Haná region, with Olomouc serving as its geographical, historical, and economic center (European Union 2007). One of the first mentions of this cheese was in 1452, as reported by Kux (1943); another mention was also found in Olomouc's state list in 1583 (A. W. spol. s r. o. 2024; Hybšová 2020). The manufacturing process, from the procurement of raw materials (skimmed sour curd) to packaging, must be executed within the Haná region (European Union 2007).

According to the EU regulation, Olomoucké tvarůžky is classified as a skimmed cheese that is ripened under a smear layer. This process results in its typical smell and savory, pungent flavor, which develops due to deep proteolysis by the microflora during the ripening process (European Union 2007). The flavor profile of the product ranges from mild to very penetrating, depending on the degree of ripening (European Union 2007; Kružíková and Lakdawala 2024).

The surface of Olomoucké tvarůžky exhibits a golden‐to‐orange smear, while its body is semi‐soft to soft and has a cohesive consistency and a lighter core (A. W. spol. s r. o. 2024; European Union 2007). Typically, these cheeses are shaped like disks, rings, or sticks, weighing about 20–30 g each. These products are also available in irregular pieces and can be consumed as table cheese or as an ingredient in numerous traditional and modern Czech cuisine dishes (A. W. spol. s r. o. 2024; Kružíková and Lakdawala 2024). Concerning Olomoucké tvarůžky composition, the PGI specifies that fat content must not exceed 1% and the dry matter content must be between 34% and 38% (European Union 2007).

**FIGURE 2:**
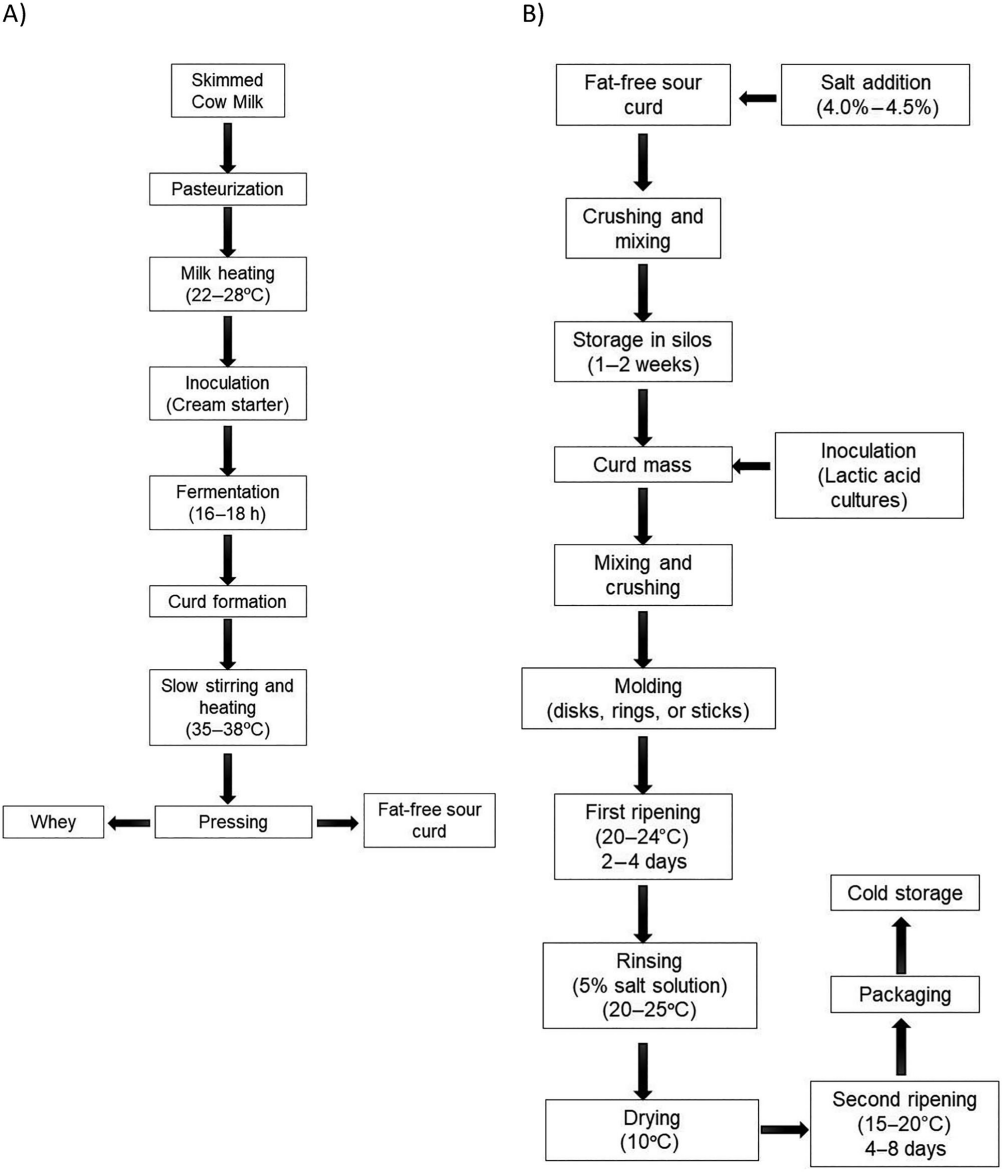
Schematic process of Olomoucké tvarůžky cheese production. (A) Industrial cottage or sour curd production; (B) Olomoucké cheese production.

Figure 2 shows the basic steps involved in the production of Olomoucké tvarůžky. Olomoucké tvarůžky is a cow's milk cheese that is produced without the addition of rennet, colorants, aromas, or stabilizers. Unlike most cheeses, its production begins not with milk as raw material but with a fat‐free industrial sour curd (also called Tvaroh) as raw material (European Union 2007; Kalab and Palo 1987; Obermaier and Čejna 2013).

The curds used as raw material for Olomouc cheese production follow its process, as shown in Figure 2A. The curd or cottage is produced with pasteurized cow skimmed milk by the precipitation induced by the lactic acid produced by dairy microorganisms without the addition of rennet. First, the milk is heated at 22–28°C prior to milk inoculation (Obermaier and Čejna 2013). It has been reported that the use of a cream culture includes Gram‐positive cocci (*Micrococcus*), coryneform bacteria (*Brevibacterium*) and yeasts (*Candida*; Obermaier and Čejna 2013), or a starter composed of lactic acid (*Lc lactis* subsp. *cremoris*, *Lc. lactis* subsp. *lactis*) and flavor‐producing bacteria (citrate‐positive lactococci or *Leuconostoc mesenteroides* subsp. *cremoris*; Fox et al. 2017). After 16–18 h of fermentation and curd formation, the curd is stirred slowly and heated at 35–38°C to solidify and promote whey separation. This is followed by a pressing step until the targeted dry matter (DM) is obtained. The curd must have a homogenous, firm, and cohesive consistency; the color is white, with a slight aroma and sour taste. Some cavities may be present but without syneresis. This curd has a crumbling structure with large, tough grains; additionally, it has biological activity and a high level of acidity (European Union 2007). Fox et al. (2017) reported that dry‐curd cottage cheese has less than 20% DM, 0.4% fat, and 17.3% protein, while low‐fat cottage has 17.5% DM, 1.0% fat, and 12.4% protein. The rapid acidification while agitating at a high temperature results in the precipitation of the casein rather than its gelation and conducts to a rapid aggregation with a high degree of fusion of the casein particles and shrinkage, forming less porous and hydrated aggregates (Fox et al. 2017). Regarding the evaluation of the curd quality, Němečková et al. (2018) mentioned that since it is contractually agreed between the supplier and the client, the information is not accessible to the rest of the general public. Pavlák's ripening test is used to evaluate the suitability of cottage cheese for producing cheese curds, as mentioned by these authors. On the other hand, Němečková et al. (2018) reported, for industrial curds, a total number of 9.0 × 10^3^–6.0 × 10^5^ for alkaligenic microorganisms, < 1 × 10^3^–2.0 × 10^6^ of total acidogenic microorganisms, < 1 × 10^1^ of Gram‐negative aerobic bacteria, and < 1 × 10^1^–4.5 × 10^3^ of Enterobacteriaceae, while for yeast and molds, they reported 6.0 × 10^2^–5.0 × 10^4^ and < 1 × 10^1^–< 1 × 10^3^, respectively, and finally, 1 × 10^4^–3.8 × 10^6^ of proteolytic microorganisms.

For cheese production, different procedures are described. Figure 2B shows the basic steps of the Olomouc cheese production process described in the PGI (European Union 2007). The process begins with the storage of the curds for a period of 2–4 weeks (short‐time) or up to 1 year (long‐storage). Long‐storage curds must be mixed with short‐time–stored curds in a proportion of a maximum of one‐third, depending on curd physicochemical changes induced during the storage time, to obtain an acidity of 120–140°SH (Spxhlet Henkel degrees). The next step is the addition of salt to adjust it to 4.5%, and if necessary, water to adjust dry matter to 32%–34% (Chromečková 2010; Hybšová 2020; Kalab and Palo 1987). Then, the treated curd is stored in silos under compact conditions without air until it achieves the desired homogenization (European Union 2007). Next, the curd is inoculated with a mixture of *Pedioccocus acidilactici* (*PA*), *Candida valida*, and *Brevibacterium linens* (Chromečková 2010). Afterward, the soft curd is crushed and molded into the desired shapes (disks, rings, or sticks), size, and weight with an extruder and cut with a wire. Next, the molded products are placed on grids and transferred for the first ripening to rooms, where they are dried at a controlled temperature (20–24°C) for 2–4 days (European Union 2007; Kalab and Palo 1987).

The cheeses are subsequently rinsed or washed with a 5% salt solution (Hybšová 2020) using individual cheese racks and upper and lower water curtains at 20–25°C. This helps maintain the warmth needed for the bacteria, preventing cooling (Hybšová 2020). After washing, the cheeses are allowed to dry at 10°C for several hours (Hybšová 2020) to prepare for the second ripening stage. During this stage, the cheeses are ripened at 15– 20°C for a period of 4–8 days (Chromečková 2010). This process leads to the development of a golden‐yellow smear on the surface, resulting from the growth of aerobic proteolytic microflora which contribute to the cheese's flavor, taste, and color (European Union 2007). Finally, the cheeses are packaged in polypropylene films, allowing ripening to continue in cold storage.

It has been reported that the most significant microorganisms found on the surface of cheese during the drying process include yeasts such as *Candida crusei* and *Can. mycoderma, Can. moravika*, *Torulopsis candida*, *Tor. olomucensis*, *Tor. lactis*, and molds such as *Oospora lactis* and *O. casei* (Hybšová 2020; Pachlová et al. 2013). These microorganisms are responsible for the oxidation process on the cheese surface, where lactic acid is oxidized to pyruvic acid, which is then decomposed into carbon dioxide and water. The washing step removes these oxidizing microorganisms, creating a suitable environment for aerobic microorganisms. During the second ripening phase, conditions become favorable for the proliferation of aerobic bacteria. This bacteria includes *Brevibacterium linens* (which causes the surface to turn orange) and other non‐starter lactic acid bacteria (NSLAB). Additionally, bacteria like *Lactobacillus casei*, *Lb. Lactis*, and *Lb. helveticus* are added, contributing to the development of cheese's characteristic features (Hybšová 2020; Pachlová et al. 2013).

#### 2.1.2 Compositional and Biochemical Characteristics

The existing literature provides only limited information about the nutritional composition of this kind of cheese, and details are not always fully specified. Some authors reported only two parameters, while others included up to five. The dry weight content values ranged from 33% to 39.15% (Kabelová et al. 2009; Strnadová et al. 2012), fat content in dry matter (FCDM) values are within 0.2%–0.9% (Čurda and Štětina 2020; Kabelová et al. 2009), and for NaCl, values go from 1.8% to 6.94% (Čurda and Štětina 2020; Standarová et al. 2010, 2012).

This cheese has an effective proteolytic apparatus that facilitates the release of free amino acids (FAA), precursors of aroma compounds, and biogenic amines. Table 1 shows the FAA profile that has been reported for this cheese (Kabelová et al. 2009; Pachlová et al. 2015). Kabelová et al. (2009) reported a total of 70.6 g FAA/kg of cheese in commercial Olomouc cheeses, where the most abundant FAA were Glu, Pro, and Leu, followed by Phe and Asp (Table 1). Conversely, Pachlová et al. (2015) examined the FAA formation throughout ripening and storage after packaging. They reported lower levels, 30.3 g/kg of total free amino acids at the end of the storage evaluation. Lys, Gln, and Glu were the most abundant FAA for all the sampling points, followed by Leu, Asn, and Val (Table 1).

**TABLE 1:** Free amino acids in different Czech cheeses.

Cheese	Concentration of free amino acids (g/kg)																					Ref.^a^
	Asp	Cys	Cit	Ser	Glu	Gly	Gln	His	Arg	Asn	Thr	Ala	Pro	Tyr	Val	Met	Lys	Ile	Leu	Phe	Total	
Olomoucké tvaruzky^b^	5.3			1.2	15.6	1.4		1.2	1.2		1.8	1.4	8.4	1.5	5.1	1.7	6.7	3.5	7.8	6.8	70.6	a
	≈0.65	≈0.60	≈0.20	≈0.40	≈2.9	≈0.60	≈3.0	≈1.05	≈0.15	≈2.60	≈0.65	≈1.0	≈1.98	≈0.60	≈2.1	≈0.98	≈3.35	≈1.45	≈2.60	≈1.7	30.3	b
Niva	10.1			3.4	37.2	2.3		1.8	5.3		2.5	2.6	18.1	3.8	7.4	1.3	9.9	3.2	10.8	8.8	128.5	a
Blat'acké zlato	7.2			2.7	18.1	3.0		2.7	2.4		2.4	2.9	10.2	2.1	7.7	2.8	7.3	3.5	7.7	2.1	84.8	a
Eidam	6.1			1.4	14.7	1.4		1.3	1.8		4.2	2.1	6.7	2.9	7.3	2.4	6.8	2.3	6.9	4.8	73.1	a
Camembert	5.4			1.1	12.8	0.6		0.7	1.6		1.5	2.8	7.2	0.9	3.0	0.3	5.8	0.9	5.8	1.9	52.3	a
Vltavin	5.8			1.2	13.8	0.7		0.8	2.5		2.2	1.8	6.8	1.0	3.6	0.3	5.9	1.0	6.1	2.9	56.4	a
Piviní sýr	11.7			4.1	42.0	5.3		4.0	6.4		5.2	3.1	21.8	4.7	12.2	4.4	13.3	5.8	11.7	3.8	159.5	a
Jadel	3.3			1.4	8.8	1.2		1.2	2.1		1.7	2.5	5.5	1.7	1.8	1.9	3.2	1.6	3.2	2.1	43.2	a
Romadur	9.8			1.8	29.5	2.8		1.9	2.3		3.6	2.9	13.2	1.5	9.6	2.3	12.1	7.1	14.1	9.7	124.2	a

Abbreviations: Ala: alanine; Arg: arginine; Asn: asparagine; Asp: aspartic acid; Cit: citrulline; Cys: cysteine; Gln: glutamine; Glu: glutamic acid; Gly: glycine; His: histidine; Ils: isoleucine; Leu: leucine; Lys: lysine; Met: methionineIle; Phe: phenylalanine; Pro: proline; Ser: serine; Thr: threonine; Tyr: tyrosine; Val: valine;.

^a^ Ref: Reference: a: Kabelová et al. (2009); b: Pachlová et al. (2015).

^b^ Authors differ in concentration values and presented different FAA profiles.

This cheese is often described as having pungent or strong flavors, with notes that can be malty, flowery, fruity, or garlicky (Pachlová et al. 2013). Forty‐six aroma compounds have been reported for this cheese (Chromečková 2010; Pachlová et al. 2013). These compounds include 14 alcohols (1,3‐butanediol, 2,3‐butanediol, 2‐butanol, 2‐heptanol, 2‐methyl‐1‐butanol, 2‐methylpropan‐1‐ol, 2‐nonanol, 2‐propanol, 2‐undecanol, 3‐methyl‐1‐butanol, 4‐ethylphenol, benzyl alcohol, ethanol, and phenylethyl alcohol), four aldehydes (3‐methyl butanal, benzaldehyde, nonanal, and phenyl acetaldehyde), seven ketones (2‐butanone, 2‐heptanone, 2‐nonanone, 2‐propanone, 2‐tridecanone, 2‐undecanone, and 4‐undecanone), 10 esters (1‐undecen‐1‐yl acetate, 2‐methyl butyl acetate, 2‐methyl propanoate, 2‐methyl propyl acetate, 2‐phenylethyl‐2‐methyl propanoate, 2‐phenyl ethyl acetate, 2‐phenyl ethyl butanoate, 3‐methyl butyl acetate, ethyl acetate, and ethyl decanoate), 2 phenolic compounds (m‐cresol and p‐cresol), three sulfur compounds (dimethyl disulfide, dimethyl trisulfide, and Methanethiol), five acids (2‐methyl butanoic acid, 2‐methyl propanoic acid, 3‐methyl butanoic acid, Acetic acid, and Butanoic acid), and one terpene (limonene). Chromečková (2010) described the behavior of some compounds during ripening: 2‐butanone increased until 42 days ripening (d42) then decreased, 2‐butanol increased until d35, phenethyl alcohol decreased continuously throughout ripening, and 3‐methyl‐1‐butanol decreased drastically between d0 and d7 and then continued decreasing slightly.

On the other hand, the formation of different biogenic amines (BA) has also been previously reported (Table 2). Komprda et al. (2012) and Standarová et al. (2010) evaluated the effect of storage temperature (5 and 20°C) on the BA formation. Both authors found that the most abundant BA was tyramine, followed by putrescine and cadaverine, concluding that higher storage temperatures increased the BA content.

**TABLE 2:** Biogenic amines in Czech cheeses.

Cheese	Total content (mg/kg)	Conditions	Tyramine (mg/kg)	Cadaverine (mg/kg)	Putrescine (mg/kg)	Reference^a^
Olomoucké tvaruzky		5°C	≈380	≈150	≈250	a
		20°C	≈1400	≈1450	≈750	
	850.7	Disks	269.1	347.6	93.6	b
	973.7	Rings	196.2	360.5	251.1	
	584.4	Sticks	187.8	149.8	125.5	
	403‐516	Initial	117	124	75.8	c
		7 days at 5°C	190	446	174	
		7 days at 20°C	798	1051	344	
	26.5–69.1^b^	Rings	5.30–39.1	1.66–10.5	1.31–6.02	d
	42.1–171^b^	Cylinders	8.85–50.8	3.51–69.6	3.45–21.6	
	97–242^b^	Disks‐small	36.1–64.6	3.46–128	2.39–36.8	
	24.7–88.8^b^	Disks‐large	3.17–11.9	2.16–26.3	2.13–11.9	
	64.9–571.4	Disks	15.4–92.1	nr	5.5–73.7	e
Niva	89–1366	Core	58–875	45–235	0–117	f
		Edge	10–411	3–491	0–44	
	38.6–985.9	Different producers	2.9–337	3.0–705	2.0–61.1	g
Edam	≈350	10°C	200–250	40–50	100–150	h
	≈800	16°C	400–500	50–105	150–225	
		Different conditions	50–125	5–15	70–160	i
Camembert	14.5	ns	1.65	0	0.92	b
Romadur	186	ns				j
	687.3	ns	182.6	271	161.6	b
Bryndza	73.2–222.2	Farmer market	34.6–107.4	16.5–42.6	22.1–60.9	k

Abbreviation: nr: no reported.

^a^ a: Komprda et al. (2012); b: Pojer (2011); c: Standarová et al. (2010); c: Samková et al. (2025); e: Cwiková and Franke (2019); f: Komprda et al. (2008); g: Standarová et al. (2009); h: Pachlová et al. (2012); i: Buňková et al. (2010); j: Pelikánová and Křížek (1995); k: Buňková et al. (2013).

^b^ Sum of Putrescine, Cadaverine, Tyramine, and Histamine.

The impact of the cheese shape on BA formation was reported by Pojer (2011). According to this author, cheeses shaped such as rings contained a total of 973.7 mg BA/kg, while disks and sticks had 850.7 and 584.4 mg BA/kg, respectively, with cadaverine, putrescine, and tyramine as the most abundant BA (Table 2).

The effect of the sampling period, storage, and shape on BA content has been recently evaluated (Table 2). Samková et al. (2025) assessed the BA content of four different‐shaped cheeses produced in different years. The study revealed significant variations in the sum of the four main BA (putrescine, cadaverine, histamine, and tyramine) and the individual BA values across different production years and cheese shapes; for the sampling time, no significant differences were detected for the sum of BA and tyramine. The authors reported that the sum of the four main BA was higher in disks‐small shaped (97–242 mg/kg) than in the other shapes, while cylinders (42.1–171 mg/kg), disk‐large shaped (24.7–88.8 mg/kg), and rings (26.5–69.1 mg/kg; Table 2). For all the analyzed cheeses, tyramine was the most abundant BA, followed by cadaverine and putrescine (Table 2).

### 2.2 Jihočeská Niva and Jihočeská Zlatá Niva

#### 2.2.1 PGI and Production Process

Jihočeská Niva and Jihočeská Zlatá Niva are cheeses produced in the Český Krumlov plant in the Czech Republic since 1951, with the same production process. In 2020, both cheeses were granted a PGI in 2010 (European Union 2010a, 2010b). The PGI Jihočeská Niva and Jihočeská Zlatá Niva cheeses are produced in the southern Bohemia region (Jihočeský kraj), which is one of the less polluted areas in the Czech Republic. The pastures are located in the protected zones of Novohradské Hory (Nové Hrady Mountains), Blanský les (Blanský Forest), and Šumava (Bohemian Forest), giving a positive influence on milk flavor (European Union 2021a, 2021b).

According to EU regulations, both cheeses are made with cow's milk from a protected geographical area, and the entire production process, ripening, and packaging must occur within this designated area.

Jihočeská Niva cheese has a mild consistency, is crumbly when spread, and is evenly ripened (European Union 2021a); Jihočeská Zlatá Niva cheese presents the same characteristics but is milder than Jihočeská Niva (European Union 2021b). Both cheeses must be cylindrical in shape with a diameter of 180–200 mm, a height of about 10 cm, and an approximate weight of 2.8 kg (European Union 2021a, 2021b). Their surface could present a cream to light brownish color, and treatments such as scraping or washing are permitted; the bluish‐green mold could cover part of this surface without being detrimental, while the internal part could present an off‐white to cream color and regular green to bluish‐green marbling due to the molds in the paste and the pierce marks (European Union 2010a, 2021b).

Regarding the composition of the cheeses, the PGI stipulates that both cheeses must contain 52% dry matter. However, there are differences in other compositional parameters. Jihočeská Niva must have a fat content in dry matter of 50% (with limits of 50% to less than 55%) and a salt content of 2.5%–5.5% (European Union 2021a). In contrast, Jihočeská Zlatá Niva is required to have an FCDM of 60% (limits 60% to <65%) and salt content of 2.5%–6.0% (European Union 2021b). Both cheeses are available for sale whole, in halves, or portions of various weights.

**FIGURE 3:**
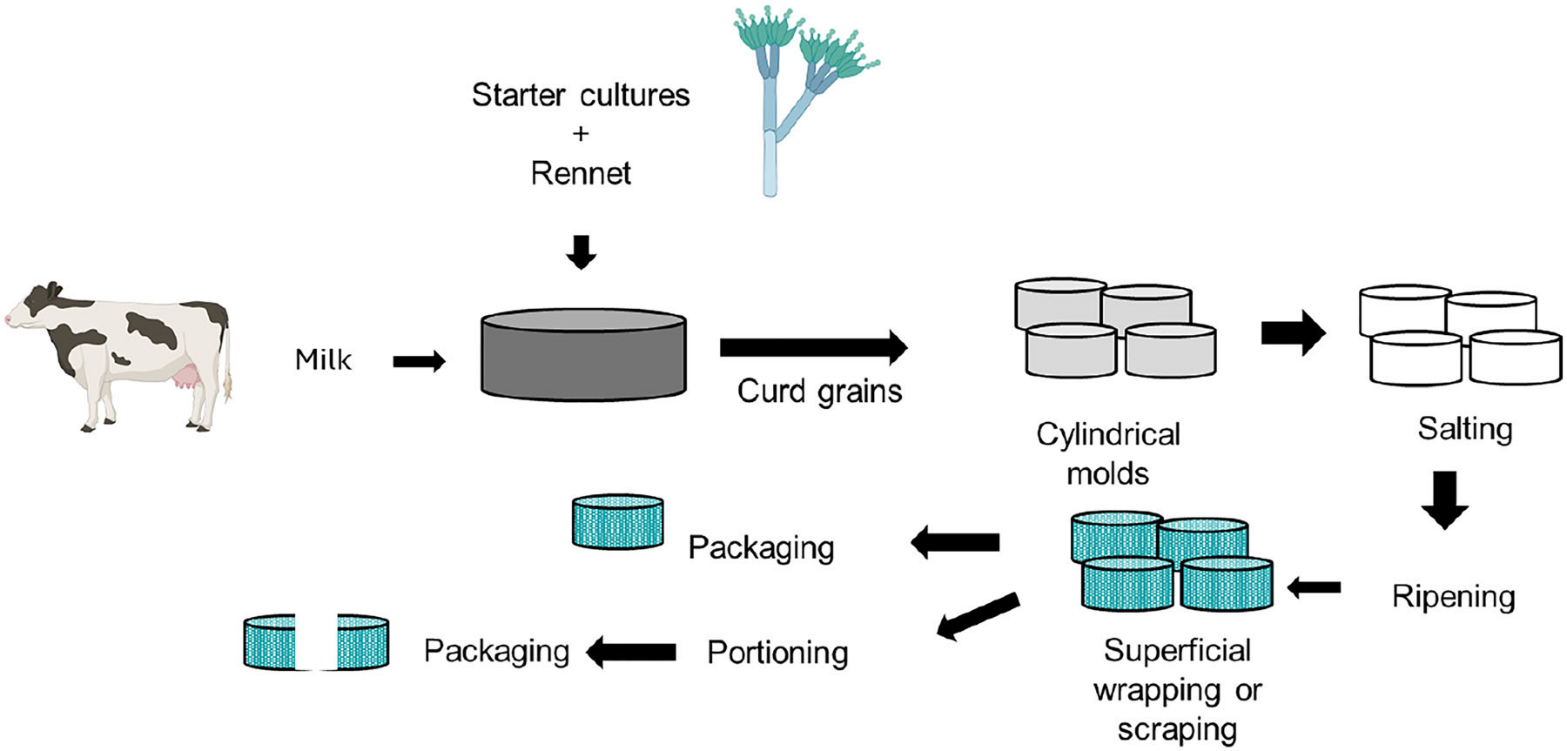
Schematic process of Jihočeská Niva and Jihočeská Zlata Niva cheeses production (European Union 2021a, 2021b).

Figure 3 shows the basic steps for Jihočeská Niva and Jihočeská Zlatá Niva production. Only the milk produced within the designated protected area can be used for both cheeses, and the same procedure is followed. However, there is a difference in the milk fat content depending on the kind of cheese. For Jihočeská Niva, milk fat content must be 3.45% ± 0.15%, while for Jihočeská Zlatá Niva, it must be 5.35% ± 0.15% (European Union 2021a, 2021b). As a first step, starter cultures are added to the milk. Generally, the starter cultures include strains of *Lactococcus lactis*, subsp. *lactis*, and *L. lactis*, subsp. *cremoris* (Komprda et al. 2008) and strains of *Penicillium roqueforti* mold PY or PV, CB, or PR1 (up to PR4), but the use of other strains of *P. roqueforti* produced by other producers is permitted if they present properties corresponding to the listed strains (European Union 2021a, 2021b). After rennet addition, the curd grains are transferred to cylindrical molds, and gravity drains the whey. The salting process can be performed in one of two ways: (a) cheeses can be immersed in a brine bath and then rubbed with coarse‐grained salt or (b) salt can be applied to the surface. The cheeses are then ripened in cellars for approximately 4 weeks under controlled temperature and humidity. Ripened cheese surface is washed or scraped; if this is the case, they are cut and portioned, but in all cases, the packaging is performed directly in the production facility using aluminum foil or a special oxygen‐permeable film (European Union 2021a, 2021b).

#### 2.2.2 Compositional and Biochemical Characteristics

Regarding the Olomoucké tvarůžky cheese, the literature only provides incomplete information about the nutritional composition of this kind of cheese, and it is not always completely specified which of the two kinds of cheeses is reported. For the Dry Weight content values, the range goes from 52% to 55.1% (Čurda and Štětina 2020; Kabelová et al. 2009), 50% for FCDM (©NutriDatabaze.cz 2025). It has been reported 20 g of protein/100 g, 1.3 g of carbohydrates/100 g, 28.9 g of total fat/100 g, 1833 mg Na/100 g, and 4.7% NaCl (©NutriDatabaze.cz 2025). In addition, Altangerel et al. (2011) reported 2.112 ± 0.027 mg pantothenic acid, 0.992 ± 0.048 mg nicotinamide, and 0.129 ± 0.013 mg pyridoxine/100 g of cheese; these values are similar to the values reported by ©NutriDatabaze.cz (2025).

The profile of the free amino acids (FAA) for commercial Niva cheese (without specification of the kind of Niva) has been reported by Kabelová et al. (2009; Table 1). This author reported a total of 128.5 g FAA/kg of cheese, with Glu, Pro, Leu, and Asp being the most abundant.

The aroma compounds of Niva cheeses have also been evaluated. Vítová et al. (2006) followed the aroma compounds in Niva cheese (50% dry matter, 55% fat in dry matter) by SPME‐GC throughout 60 days of ripening. They reported 54 compounds identified: 18 alcohols (2‐methylpropan‐1‐ol, 3‐methylbutan‐1‐ol, butanol, dodecan‐1‐ol, ethanol, heptadecan‐1‐ol, heptadecan‐2‐ol, heptan‐2‐ol, hexadecan‐2‐ol, methanol, nonan‐2‐ol, oct‐1‐en‐3‐ol, octan‐1‐ol, pentan‐1‐ol, pentan‐2‐ol, phenyl ethanol, propan‐1‐ol, and propan‐2‐ol), 11 ketones (3‐hydroxybutan‐2‐one, acetone, butan‐2‐one, butan‐2,3‐dione, heptan‐2‐one, propan‐2‐one, pentan‐2‐one, nonan‐2‐one, 8‐nonen‐2‐one, decan‐2‐one, and undecan‐2‐one), 10 fatty acids (acetic, benzoic, butanoic, capric, hexanoic, isobutanoic, isopentanoic, myristic, palmitic, pentadecanoic, and propionic acids), five aldehydes (benzaldehyde, ethanal, hexanal, phenylacetaldehyde, and propanal), four sulfur compounds (benzothiazole, dimethyl disulfide, dimethyl sulfide, and dimethyl trisulfide), three esters (ethyl‐acetate, phenyl ethyl‐acetate, and pentyl benzoate), and three hydrocarbons (heptadecane, heptane, and pentadecane).

On the other hand, the free fatty acids profile was also evaluated by Vítová et al. (2004). These authors identified and followed the fatty acid profiles of Niva cheese over the course of 60 days of ripening. They reported the identification of 30 fatty acids with the highest levels detected for palmitic, oleic, myristic, stearic, and capric acids (in decreasing concentration order).

BA are formed from the FAA in this cheese, as in other blue cheeses. Komprda et al. (2008) evaluated the differences in the content of BA in Niva cheese depending on the cheese section and the season (Table 2). While no significant differences were reported among the analyzed cheeses, higher concentrations of BA were found in the cheese core than in the cheese edge. Standarová et al. (2009) evaluated BA on cheeses from different producers, seasons, and two cheese sections (Table 2). They mentioned that the BA content and the profile exhibited variability between the producers. For a given producer, higher levels of total BA were observed in June cheeses, with higher concentrations in the core than in the edge.

## 3 Non‐Protected Czech Cheeses

In the Czech Republic, a variety of cheeses are produced and sold in local markets; however, none of them have received PGI status. Most of these cheeses are similar to other European varieties and often lack a unique or distinctive name; however, they have been produced in the country for several decades.

### 3.1 Blat’ácke Zlato Cheese

The first registers of the production of Blaťácké zlato cheese production date back to 1939. It is a soft cheese, similar to the Italian Bel Paese cheese. Pasteurized cow's milk is used in its production, and one of its characteristics is that whey drainage is achieved by gravity with occasional mold rotation. Salting is carried out after molding by submerging the molds in the brine, followed by a ripening period. The cheeses undergo a ripening process at low temperatures, which limits their microbial growth; therefore, during ripening, the changes are produced mainly by the rennet enzymes that act throughout the mass for several months (Obermaier and Čejna 2013). During ripening, a small smear layer is formed, and it slightly participates in the ripening process (Obermaier and Čejna 2013), but now, the modern procedure includes covering the surface with a pigment such as annatto, giving this yellowish color to the surface. At about 20 days of ripening, the whole cheeses are packaged in a heat‐shrink plastic film, with or without protective substances, to continue the ripening process for an additional 4–6 weeks. When cheese passes to the market, it contains about 53.8% dry matter and 17.5% fat (Čurda and Štětina 2020).

Despite its long‐standing presence in the Czech market, scientific information regarding this cheese remains limited, inaccessible, or outdated. Some efforts to optimize the production process were made years ago. Ledabyl (1956) focused on evaluating the starter cultures; this author reported that the application of “steaming” gave better results at 38–40°C and 100% relative humidity (RH) during 3 h, giving an acidity of 40–60°SH. Additionally, Ledabyl (1957) mentions that the optimal results were achieved with a streptococcal nisin culture, and the less desirable results were obtained with a mixture of *L. casei* and *Str. thermophilus* culture. Dolezalek et al. (1973) evaluated the effect of milk fat content, pasteurization conditions, salt addition, different starters, and even different microbial rennet in the final quality of the cheeses. However, they mentioned that they found changes in the nitrogen fraction, free amino acids, and rheological properties, but values are unavailable. There are limited reports about the surface microflora of this cheese. On the surface of Blat’ácke zlato cheeses, Hanušová, Dobiáš, and Voldřich (2012) isolated different strains of microorganisms that cause spoilage on cheese surface, among them, the authors reported the presence of *Debaryomyces hansenii*, *Penicillium camembertii*, *Penicillium chrysogenum*, *Penicillium expansum*, *Penicillium brevicompactum Penicillium crustosum*, *Penicillium commune*, *Cladosporium herbarum*, *Cladosporium cladosporioides*, *Penicillium* sp. (subg. *Furcatum*, series *Citrina*).

Recent studies have focused on evaluating the films utilized in these cheeses (Hanušová et al. 2009, 2012). These authors assessed various film materials, such as polyethylene (LDPE and PVC) coated with active substances (nisin and/or natamycin), for active packaging to preserve or even enhance the quality of this cheese during storage.

Regarding the cheese composition, it has been reported a 51%–53.8% of dry matter (Čurda and Štětina 2020; Kabelová et al. 2009), protein content of 20.9 g/100 g, fat 27.5 g/100 g (©NutriDatabaze.cz 2025) with 48% of FDM (Čurda and Štětina 2022; Kabelová et al. 2009), carbohydrates 1.9 g/100 g, 840 mg Na/100 g, and 2.1 g/100 g NaCl (©NutriDatabaze.cz 2025). Additionally, a total of 84.8 g/kg of free amino acids in commercial cheese have been reported by Kabelová et al. (2009), where the most abundant were Glu, Pro, Val, and Leu (Table 1).

### 3.2 Dutch‐Type Natural Cheese (Eidamska Cihla)

Eidam cheese is produced using a process similar to other Dutch cheeses, where the curd is cut into cheese grains the size of a pea, stirred for a long time, and part of the whey is drained. Next, hot water is added to reach a low heating temperature of around 37°C, continuing with an intensive stirring. Subsequently, curd grains are transferred to perforated pressing vats for whey drainage. These cheeses are salted for 1–2 days. The ripening lasts at least 1 month and up to 2 years (Obermaier and Čejna 2013).

It has been reported between 53% and 61% of dry matter and 30%–45% of FDM for Czech Eidam cheese and 3.90% ash content (Altangerel et al. 2011; Čurda and Štětina 2022; Kabelová et al. 2009). Altangerel et al. (2011) reported 0.050 ± 0.017 mg nicotinamide, 0.523 ± 0.032 mg pantothenic acid, and 0.077 ± 0.011 mg pyridoxine/100 g. Koreňovská and Suhaj (2008) evaluated the mineral content of Emmental and Eidam‐type cheeses, reporting the following values: Cu 1.1775 mg/kg, Ca 8310.5 mg/kg, K 1032 mg/kg, Mg 412.5 mg/kg, Na 6675 mg/kg, Ba 2.755 mg/kg, Cr 0.2655 mg/kg, Hg 0.00775 mg/kg, Mo 0.043 mg/kg, Mn 0.2995 mg/kg, Ni 0.27 mg/kg, and V 0.1855 mg/kg; in general, the values were within the mean values obtained for European cheeses.

On the other hand, Kabelová et al. (2009) found 73.1 g FAA/kg of cheese; the Glu was the most abundant FAA with almost twice the concentration of the next most abundant, Val, Leu, and Lys (Table 1). Pachlová et al. (2012) reported a total free amino acid content of about 14 g/kg of cheese at 112 days for control cheeses and 56 days for accelerated ripening cheeses.

The effect of salt reduction on the sensory attributes of Dutch‐type cheeses has been evaluated before (Grégrová et al. 2021; Němečková et al. 2023). The impact of salt reduction was assessed following the evolution of 11 volatile compounds (ethanol, diacetyl, 2‐butanone, 2‐butanol, acetic acid, acetoin, hexanal, butyric acid, 2,3‐butanediol, hexanoic acid, and octanoic acid) through ripening; however, there are not reported significant effects associated with the time in brine, that is, salt content (Grégrová et al. 2021; Němečková et al. 2023).

As with other cheeses, the ripening temperature is a critical factor in the production of biogenic amines. Pachlová et al. (2012) observed that ripening at 16°C produced a significant increase in total BA content, passing from 350 mg BA/kg at 10°C to 800 mg/kg at 16°C (Table 2), affecting differently the individual BA. While tyramine exhibited a similar trend, cadaverine and putrescine showed different increases (Table 2). They mentioned that cheeses ripened at higher temperatures presented higher concentrations of biogenic amines at 56 days compared to control cheeses at 112 days.

### 3.3 Hermelin

This cheese was initially sold under the name Camembert, and records have shown its price since 1923 (Kopáček et al. 2007). Following the registration of the name Camembert as a protected cheese, the former director of the largest producer factory proposed the name “Hermelin” to distinguish the Czech cheese (Kopáček et al. 2007). Hermelin is primarily associated with the white fur coat made from ermine winter skins. This white mold surface cheese is a camembert‐type cheese produced principally in Sedlčany in Central Bohemia (Kopáček et al. 2007).

Given the similarity of the production process to that of Camembert cheese, the starters used must be of the same kind. For Camembert, it has been reported that O, L, or DL mesophilic mixed cultures are used as starters, and *P. camemberti*, *K. lactis*, *G. candidum*, and *B. linens* are secondary starters (Fox et al. 2017). Regarding its composition, it has been reported a 45%–49% dry matter (Altangerel et al. 2011; Čurda and Štětina 2020; Kabelová et al. 2009), 22.4 g protein/100 g, 22.3 g of fat/100 g, 1.2 g of carbohydrates/100 g, 3.8 g of ashes/100 g, 1112 mg Na/100 g, and 2.8 g salt/100 g (©NutriDatabaze.cz 2025). Altangerel et al. (2011) reported 0.755 ± 0.072 mg nicotinamide, 0.460 ± 0.031 mg pantothenic acid, and 0.140 ± 0.036 mg pyridoxine/100 g.

Despite its resemblance to Camembert, this cheese has been the subject of several studies, focusing on its evaluation. Recent studies have been developed to evaluate and compare eight different *Penicillium nalgiovense* strains with two *Penicillium camemberti* strains in the production of free amino acids and sensory characteristics (Mrázek et al. 2016). The evaluation revealed that certain *P. nalgiovense* strains demonstrated high proteolytic activity, while others, exhibiting lower proteolytic activity, yielded superior sensory outcomes. For this cheese, a total of 52.3 mg of FAA/kg has been reported by Kabelová et al. (2009), with Glu being the most abundant, followed by Pro, Lys, and Leu (Table 1).

On the other hand, Vítová et al. (2007) evaluated the volatile compounds in Hermelin, Block Hermelin, and premium white surface mold cheeses by SPME‐GC. They reported the identification of 31 compounds: 11 alcohols (2‐methyl propanol, butanol, ethanol, heptan‐2‐ol, methanol, octan‐1‐ol, oct‐1‐en‐3‐ol, pentan‐2‐ol, phenyl ethanol, propan‐1‐ol, and propan‐2‐ol), seven ketones (3‐hydroxybutan‐2‐one, butan‐2‐oneundecan‐2‐one, butan‐2,3‐dione, pentan‐2‐one, nonan‐2‐one, and propanone), five fatty acids (2‐methyl propanoic acid, 3‐methyl butanoic acid, butanoic acid, ethanoic acid, and hexanoic acid), three aldehydes (ethanal, phenylacetaldehyde, and propanal), two esters (ethyl‐acetate and phenyl ethyl‐acetate), two sulfur compounds (dimethyl disulfide and dimethyl trisulfide), and one hydrocarbon (heptane). Their concentration and proportion differed for the Hermelin, Block Hermelin, and premium cheeses.

Respecting the BA, Pojer (2011) reported only 14.5 mg total BA/kg. These authors did not find cadaverine, but they reported low values of tyramine and putrescine (Table 2).

### 3.4 Balkansky Syr (Balkan Cheese)

Balkansky syr is a white‐brined, semi‐hard, salty cheese produced from cow's milk, analogous to Feta cheese. For Balkan cheese, the pressing process is not used, and after forming and fermentation, they are placed in brine, where they remain until consumption (Obermaier and Čejna 2013). The product contains about 41.5% of dry matter and 50% FDM (Čurda and Štětina 2020, 2022; Lazárková et al. 2021); 14.7 g of protein/100 g, 20.5 g of fat/100 g, 1.2 g of carbohydrates/100 g, 1764 mg Na/100 g, and 4.4 g salt/100 g (©NutriDatabaze.cz 2025). Altangerel et al. (2011) reported 0.018 ± 0.006 mg pantothenic acid and 0.006 ± 0.002 mg pyridoxine/100 g. For free amino acids, a total amount within 0.05%–3.29% w/w was reported by Lazárková et al. (2021).

Concerning the cheese flora, it has been reported that for feta‐type cheeses such as Balkan cheese, two yeast species capable of fermentation (K. lactis and Pichia fermentans) are part of the typical microbiota. Additionally, *D*. *hansenii* has been reported to be on the brine (Fröhlich‐Wyder et al. 2019).

Recently, some research has been conducted to evaluate the effect of prolonged storage on canned Balkan cheese (Lazárková et al. 2021). These authors conclude that microbiologically canned Balkan cheeses were stable when stored for up to 6 months at 22°C. However, other compositional and organoleptic characteristics were stable at this temperature for only 3 months.

### 3.5 Vltavin

This traditional Czech blue‐veined cheese combines white surface and inner blue molds. It has a creamy and melting consistency, a lightly sour and milky taste with a mushroom trace (Obermaier and Čejna 2013). This cheese presents 53% dry matter, 57% FDM, and 3.35% ashes (Altangerel et al. 2011; Kabelová et al. 2009). Altangerel et al. (2011) reported 1.148 ± 0.058 mg nicotinamide, 0.1.940 ± 0.054 mg pantothenic acid, and 0.311 ± 0.022 mg pyridoxine/100 g. About FAA, Kabelová et al. (2009) reported 56.4 mg/ kg, with Glu as the most abundant, followed by Pro, Leu, and Lys (Table 1).

### 3.6 Gran Moravia

This cheese is produced in the Moravia region, following the Italian tradition of producing aged cheeses, such as Grana Padano or Parmigiano Reggiano. This is a hard cheese, with 30% fat, produced from Moravian cow's milk (in an eco‐sustainable chain) without the use of animal rennet. After its production in the Moravia region, the cheeses undergo a long ripening process in Italy. This cheese presents 31% protein and 28% fat (Li et al. 2022).

Maillard reaction products have been evaluated by Li et al. (2022). These authors reported the presence of furosine, 2‐furaldehyde, 2‐furyl methylketone, 5‐methyl‐2‐furfural, 5‐hydroxymethylfurfural, N^ε^‐carboxymethyllysine, and N^ε^‐carboxyethyllysine.

Even if this cheese is made using the traditional Italian methodology, it shows significant differences in stable isotopes of C, N, H, and S (Camin et al. 2012) and non‐target mass spectrometry analysis (Popping et al. 2017) compared with the Parmigiano Reggiano. It is important to mention that those studies focused not on Gran Moravia cheese characterization but on Grana Padano authentication.

### 3.7 Other Czech Cheeses

There is a wide variety of Czech cheeses on the market, yet there is a significant lack of scientific information about them. Some of these cheeses, such as Buda cheese (Budské sýry, Krkonoše Mountains), Nalžov cheese (Nalžovy in the Šumava Mountains), Sázava cheese, Ottawa (Polička), Knight Žumbera (West Bohemian region), and Maršov Cheese (Nové Město na Moravě), have been produced for decades, but their methods are not well documented (Kopáček 2009). Other Czech cheeses are still present in the markets; among them, we can find the Lučina, Gervais Liptov, Pivni, Jadel, Goldenburg, Romadur, Akawi, Abertam, Brynza (Ewe's milk), Koliba, Kamadet, Tizian, Gazdovsky, Ostepek (sheep's milk), and Klobučik, among others. However, database records on the subject are limited. Some examples are described below.

Lučina is a fresh and creamy cheese created in 1981, combining cottage cheese and cream. It contains 40% dry matter, 41% fat, and 1.91% ashes (Altangerel et al. 2011; Čurda and Štětina 2022). Altangerel et al. (2011) reported 0.025 ± 0.007 mg nicotinamide, 1.113 ± 0.062 mg pantothenic acid, and 0.045 ± 0.016 mg pyridoxine/100 g.

Pivni is a cow's milk, that is a full‐fat, soft, smear cheese. Originating in Germany, it was introduced in the Czech Republic in 1924. It is now completely assimilated into Czech cuisine. It is also referred to as beer cheese; however, it does not contain beer but is often consumed with it or even introduced in beer. It has been reported a 40% dry matter and 40% FDM (Kabelová et al. 2009), 0.036 ± 0.007 mg nicotinamide, 0.770 ± 0.021 mg pantothenic acid, and 0.134 ± 0.026 mg pyridoxine/100 g (Altangerel et al. 2011). Regarding FAA, this cheese presented 159.5 g/kg of cheese, with Glu being the most abundant, followed by Pro, Lys, and Val (Kabelová et al. 2009; Table 1).

Jadel cheese contains 57%–57.9% dry matter, 22.5% fat, 37% FDM, and 5.8% salt (Čurda and Štětina 2020; Kabelová et al. 2009). Altangerel et al. (2011) reported 0.081 ± 0.019 mg nicotinamide, 0.307 ± 0.028 mg pantothenic acid, and 0.012 ± 0.001 mg pyridoxine/100 g. It presents 43.2 mg FAA/kg, with Glu, Pro, Lys, and Leu the most abundant (Kabelová et al. 2009; Table 1).

Romadur cheese is a soft‐smear cheese originally produced in the Bavarian region of southern Germany. Romadour cheese is produced using whole or partially skimmed cow's milk (Miller and Skinner 2012; Sanders 1953). In its production, a cream culture comprising Gram‐positive cocci (Micrococcus), coryneform bacteria (Brevibacterium), and yeasts (Candida) is used (Obermaier and Čejna 2013). The ripening process is meticulously executed in specialized cellars where precise temperature and humidity levels are maintained. During the approximately 3‐week ripening period, the cheeses are regularly turned and thoroughly washed with a salt solution to ensure optimal quality and texture (Obermaier and Čejna 2013). The changes in the surface of the cheese are produced by a ripening culture that is added by soaking or spraying the cheeses with a solution inoculated with noble yeast that causes partial proteolysis, giving a characteristic aroma and forming a slightly sticky surface with a superficial yellow‐orange smear and a typical taste and aroma (Obermaier and Čejna 2013). This cheese has been reported to have 44% dry matter content and 40% FDM (Kabelová et al. 2009). Additionally, these authors reported 124.2 g FAA/kg of Romadur cheese, with Glu, Leu, Lys, and Asp being the most abundant (Table 1). Regarding BA, it has been reported from 186 to 687 mg total BA/kg being the cadaverine the most abundant (Pelikánová and Křížek 1995; Pojer 2011; Table 2).

Abertam is a traditional Karlovy Vary farm cheese made with sheep's milk. It is characterized by an irregular spherical shape, a firm texture, and a natural rind with a yellow to orange color and a strong, acidic, and tasty flavor; the cheese is ripened for about 2 months (Sanders 1953). For the Goldenburg, it has been reported that it contains 60% dry matter and 4.20% ashes, 0.032 ± 0.006 mg, nicotinamide, 0.426 ± 0.028 mg pantothenic acid, and 0.056 ± 0.009 mg pyridoxine/100 g (Altangerel et al. 2011).

## 4 The Current Scenario

The EU cheese market is the largest in the world. In 2020, the European Union's cheese production reached 11,348,186 tons, with 44.9% of that production occurring in central European countries (FAO 2024). In 2020, the production of Czech cheese accounted for 1.8% of EU production and 4% of Central European production (FAO 2024). Additionally, cheese has provided European producers with enhanced export opportunities. In 2023, EU exports reached USD 23,774.9 million, with central European countries accounting for 20.65% of these exports (WITS 2024). In 2023, Czech cheese exports accounted for 1.29% of EU exports and 6.25% of central European exports (WITS 2024).

In the saturated EU market, consumers seek suppliers that offer unique products. The demand for artisanal cheeses made with traditional processes and linked with specific regions has increased. Consumers are interested in cheese safety, quality, and typical characteristics (Hrubá and Sadílek 2020).

Cheese quality is hard to define since it encompasses different parameters such as composition, nutritional value, sensory attributes, and safety. In today's globalized business environment, the protection of regional products has become a significant concern. In the Czech Republic, the labels “Czech Food Product” or “Produced in the Czech Republic” can be used voluntarily, provided that 100% of the unprocessed product's constituents are sourced from the Czech Republic and primary production takes place domestically (MZE 2024).

Sanitary standards are a critical component of cheese quality. Today, standardized processes and improved hygiene and manufacturing practices have enhanced the safety of Czech cheeses. A particular interest is the presence of pathogens and enterotoxin‐producing microorganisms in cheese‐processing plants and final products. Janštová et al. (2010) evaluated the safety of fresh goat cheeses produced at a South Moravia farm in different months. The authors reported low levels of *Enterobacteriaceae* (< 1.0 × 10^1^–1.5 × 10^3^ CFU/g) and enterococci identified as *Enterococcus faecalis* (< 5.0 × 10^1^–1.9 × 10^3^ CFU/g) with no positive samples for *E. coli* nor for *B. cereus* (*n* = 22); nevertheless, *S. aureus* was identified in nine samples where two of them showed higher account for it (2.5 × 10^2^ and 5 × 10^1^ CFU/g), and eight of them were PCR positive for the Staphyloccocical enterotoxins SEB, SEG, and SEI. Gelbíčová et al. (2021) evaluated the presence of *Klebsiella* spp. in a processed cheese plant by evaluating different process equipment, raw materials, and final products. Even though *K. pneumoniae* and *K. oxytoca* were detected in various process and personnel samples, they were not found in the final cheese samples. Another microorganism of sanitary interest is *Mycobacterium avium* subsp. *paratuberculosis* that has been implicated in the pathogenesis of Crohn's disease in humans. This microorganism was detected by Ikonomopoulos et al. (2005) in Czech cheeses obtained from various supermarkets. Using PCR methods, these authors reported that from the evaluated cheeses, 17% of hard cheeses (*n* = 23), 20% of semi‐hard cheeses (*n* = 5), and 0% of soft cheeses (*n* = 14) were positive for *Mycobacterium avium* subsp. *paratuberculosis*.

Another component naturally present in dairy products is benzoic acid. According to the Czech regulation, the allowed limit is 30 mg per kilogram of product. This is more strict than the limits established in other EU countries, which could reach 50 mg per kilogram of product. It should be noted that the addition of benzoic acid as a preservative is not allowed in dairy products. Hejtmánková et al. (2000) reported benzoic acid concentrations of 4.0– 8.08 mg/kg for commercial Eidam cheeses acquired in Prague.

## 5 Conclusions

The present review provides general information about Czech cheeses, emphasizing PGI Olomoucké tvarůžky, Jihočeská Niva, and Jihočeská Zlatá Niva cheeses. The review also includes information on other cheeses with potential PGI status, such as Blat'ácke zlato, Hermelín, and Vlatvin, which are widely consumed and highly regarded. The most recent and available scientific information is provided when it exists. To ensure a comprehensive understanding of traditional Czech cheeses with PGI or potential PGI, further scientific research is necessary. This research should focus on characterizing cultural practices, raw material origin, and the impact of processes on cheese quality, composition, and functional and sensory properties that give them their typical characteristics.

## Author Contributions

**Sandra Teresita Martín‐del‐Campo**: conceptualization, data curation, formal analysis, investigation, methodology, project administration, supervision, validation, visualization, writing–review and editing, writing–original draft. **Alexa Pérez‐Alva**: formal analysis, supervision, investigation, writing–original draft, writing–review and editing. **Diana Karina Baigts‐Allende**: resources, funding acquisition, writing–review and editing.

## Conflicts of Interest

The authors declare no conflicts of interest.
